# Sub-sensory vibratory noise augments the physiologic complexity of postural control in older adults

**DOI:** 10.1186/s12984-016-0152-7

**Published:** 2016-05-03

**Authors:** Junhong Zhou, Lewis Lipsitz, Daniel Habtemariam, Brad Manor

**Affiliations:** Institute for Aging Research, Hebrew SeniorLife, Roslindale, MA USA; Beth Israel Deaconess Medical Center, Boston, MA 02131 USA; Harvard Medical School, 1200 Centre Street, Boston, MA 02131 USA

**Keywords:** Stochastic resonance, Vibration, Somatosensation, Postural sway, Multiscale entropy

## Abstract

**Background:**

Postural control requires numerous inputs interacting across multiple temporospatial scales. This organization, evidenced by the “complexity” contained within standing postural sway fluctuations, enables diverse system functionality. Age-related reduction of foot-sole somatosensation reduces standing postural sway complexity and diminishes the functionality of the postural control system. Sub-sensory vibrations applied to the foot soles reduce the speed and magnitude of sway and improve mobility in older adults. We thus hypothesized that these vibration-induced improvements to the functionality of the postural control system are associated with an increase in the standing postural sway complexity.

**Method:**

Twelve healthy older adults aged 74 ± 8 years completed three visits to test the effects of foot sole vibrations at 0 % (i.e., no vibration), 70 and 85 % of the sensory threshold. Postural sway was assessed during eyes-open and eyes-closed standing. The complexity of sway time-series was quantified using multiscale entropy. The timed up-and-go (TUG) was completed to assess mobility.

**Results:**

When standing without vibration, participants with lower foot sole vibratory thresholds (better sensation) had greater mediolateral (ML) sway complexity (*r*^2^ = 0.49, *p* < 0.001), and those with greater ML sway complexity had faster TUG times (better mobility) (*r*^2^ = 0.38, *p* < 0.001). Foot sole vibrations at 70 and 85 % of sensory threshold increased ML sway complexity during eyes-open and eyes-closed standing (*p* < 0.0001). Importantly, these vibration-induced increases in complexity correlated with improvements in the TUG test of mobility (*r*^2^ = 0.15 ~ 0.42, *p* < 0.001 ~ 0.03).

**Conclusions:**

Sub-sensory foot sole vibrations augment the postural control system functionality and such beneficial effects are reflected in an increase in the physiologic complexity of standing postural sway dynamics.

## Background

Standing posture is controlled by a host of sensory inputs that continuously communicate with the musculoskeletal system via numerous spinal and supraspinal neural networks [1]. As such, the dynamics of postural sway (i.e., the motion of the body’s center of mass with respect to its base of support) are complex, as defined by a relatively high degree of correlated, fractal-like patterns over multiple temporal-spatial scales [2–5]. This physiologic complexity can be estimated using multiscale entropy (MSE) [6] or other metrics derived from complex systems theory [7]. In general, greater complexity has been linked to the capacity of the postural control system to adapt to both physical and cognitive stressors [8].

Somatosensory feedback from the foot soles is one critical source of input to the postural control system [9]. Both age- and disease-related impairments in this source of feedback have been linked to diminished complexity of postural sway fluctuations during quiet stance [10]. Moreover, the age-related reduction in postural sway complexity during “baseline” quiet standing conditions closely correlates with diminished ability to maintain postural control during a cognitive perturbation [11]. As such, we contend that augmentation of foot sole somatosensation may restore the physiologic complexity of postural control in older adults and thus, enhance one’s ability to adapt to the stressors of daily life.

One proven method of augmenting somatosensory feedback exploits the physical principle of “stochastic resonance [12].” This principle states that weak input to a non-linear system can be optimized and enhanced by the application of particular levels of random noise [13]. Specifically, by applying a noisy stimulus that is below the detection threshold of a given input, one can improve the ability of a physiologic system to detect that input. This phenomenon has been demonstrated in systems ranging from ion channels to sensory neurons [14, 15]. With respect to postural control, vibratory noise applied to the soles of the feet at 90 % of the sensory threshold increases standing postural sway complexity [16], as well as gait and mobility [17, 18] in older adults. The relationship between sub-sensory, vibration-induced increases in the complexity of postural movement under baseline quiet-standing conditions and overall improvements in postural control system function, however, has not yet been examined.

In a recent study [18], we demonstrated that sub-sensory vibrations as low as 70 % of foot-sole sensory threshold, delivered to the foot soles via custom-built shoe insoles, reduced the magnitude of postural sway when standing and improved performance in the Timed Up-and-Go (TUG) test of mobility in relatively healthy adults aged 65 years and older. Here, we conducted a secondary analysis of that study using MSE to quantify the degree of physiologic complexity contained within standing postural sway dynamics. We hypothesized that: 1) baseline postural sway complexity would be correlated with foot sole somatosensation when standing as well as system functionality as measured by the TUG test of mobility; 2) the applied sub-sensory vibrations would increase postural sway complexity in older adults when standing (i.e., return it to a normative and functional amount); and 3) the extent of increase in postural sway complexity would correlate with observed improvements in system functionality (i.e., TUG performance).

## Method

### Participants

A secondary analysis was completed on previously reported data acquired from 12 participants aged 65-90 years (age = 73.8 ± 8.1; body mass = 66.9 ± 13.4 kg; height = 1.6 ± 0.1 m). All participants provided written informed consent as approved by the Hebrew SeniorLife Institutional Review Board. Exclusion criteria included neurodegenerative disease, the inability to walk unassisted or stand for at least one minute without support, or an inability to feel the vibrations produced by the instrumented insole (see below) at maximum output.

### Vibratory insole

**Fig. 1:**
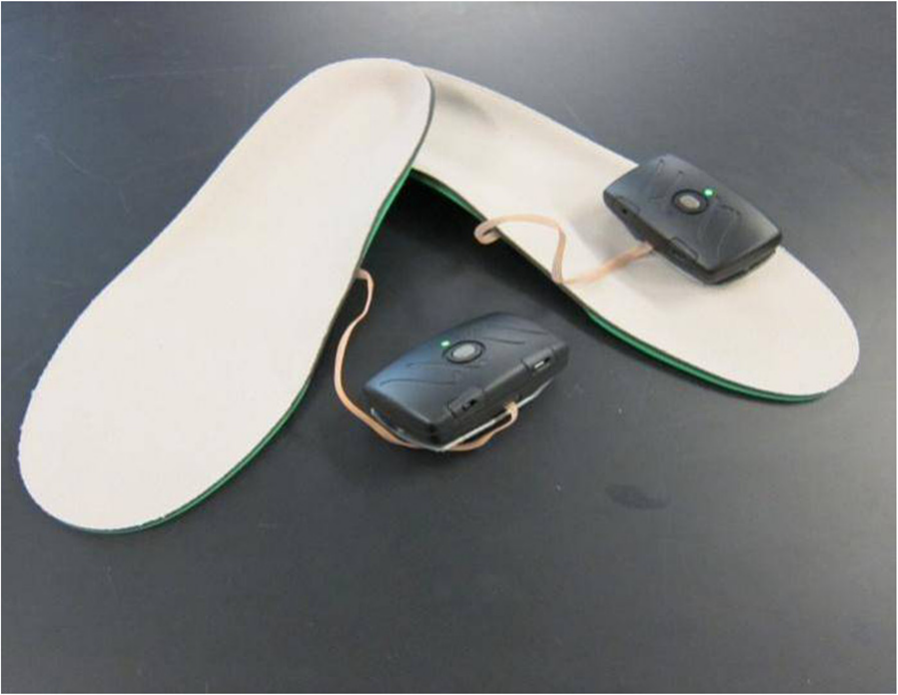
Vibratory Shoe Insoles. Stochastic resonance was delivered to the soles of the feet via controlled vibratory stimuli. Vibrations were generated by piezo-electric actuators mounted within the insoles and driven by a control unit (black box) secured to the outside top of the shoe

A full description of the vibratory insole (Fig. 1) has been previously reported [18]. Briefly, a urethane foam insole, customized with two 2.5 cm low voltage piezo-electric actuators placed 2.0 cm apart in the medial arch region of each insole, delivers white-noise-like vibrations below *100Hz* to the plantar surfaces of the feet. Multiple insole sizes were developed to ensure comfort and proper fit for each participant. Once the proper size was determined, the insoles were inserted into the subject’s footwear and each participant wore the same pair of insoles and shoes throughout all study procedures.

### Experimental protocol

Participants completed three separate study visits to test the effects of three different amplitude levels of sub-sensory vibrations, that is, 0 % (i.e., a no vibration “control” condition), 70 and 85 % of the standing vibratory sensation threshold of each foot. In order to examine the potential for adaptation to the vibration stimuli, standing postural control, foot sole vibration perception threshold and mobility were assessed during each visit within three separate testing sessions (Session 1, 2 and 3). The first session was completed in the morning and a one-hour break was provided in between each session.

The vibratory perception threshold for each foot sole was determined separately at the beginning of each visit. All thresholds were determined with the participants standing with their feet in the same position, approximately shoulder-width apart. Thresholds were obtained by a software program that automatically ramped the amplitude of insole vibration up or down. The participant was instructed to say “now” when they could, or could no longer feel the vibration. This procedure was repeated in multiple trials to narrow the boundary of sensation to a reproducible threshold [18]. On each visit, foot sole vibrations were then delivered at 0, 70 or 85 % amplitudes of the obtained vibratory perception threshold for each foot. This vibration level was set in a randomized order by study staff uninvolved with any other study procedure. As such, both participants and study personnel performing assessments were blinded to the experimental sub-sensory noise level.

### Assessment of standing postural control

Standing postural control was assessed by measuring postural sway (i.e., center-of-pressure) fluctuations at 240 Hz with a force plate (Type9286B, Kistler, Amherst, NY). The force plate was placed with its mediolateral axis parallel to the laboratory wall. Participants stood on the plate so that they were facing the wall. Tissue paper was placed on the force plate and foot placement was outlined prior to the first trial. This outline was then used throughout the study to ensure consistent foot placement across trials. During each session, four 60-s trials were completed in each of two different experimental conditions: eyes open and eyes closed. Trial order was randomized. Participants were instructed to “stand as still as possible” prior to each trial. Before each eyes-open trial, participants were also instructed to visually focus on a target “X” placed on the wall in front of them at eye-level. The position of their feet on the force plate was measured and remained the same across all the trials.

**Fig. 2:**
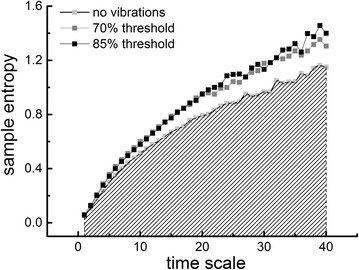
Representative multiscale entropy (MSE) curves generated from the mediolateral postural sway time-series of a single participant during eyes-open standing. For each timeseries, sample entropy was calculated and plotted as a function of time-scale (ranging from 4 to 160 ms). Postural sway complexity was defined as the area under this multi-scale entropy curve, which has been illustrated by gray slashes for the “no vibration” control condition. For this participant, sample entropy was noticeably higher across multiple time scales when they stood with sub-sensory vibrations applied to their feet, as compared to standing with no applied vibration. As such, postural sway complexity was greater (i.e., more area under the multi-scale entropy curve) when vibrations were applied at both 70 % (complexity = 33.91 units) and 85 % (complexity = 34.61 units) of threshold, as compared to the control condition (complexity = 28.9 units)

To quantify postural sway complexity, MSE analysis was completed on both the AP and ML center of pressure (COP) time-series. Prior to calculation of MSE, empirical mode decomposition (EMD) was used to remove high-frequency noise and low-frequency trends [10, 19]. Specifically, fluctuations at frequencies over 20 Hz were removed, as they are unlikely to reflect physiologically-meaningful control processes [19] and thus the noise from the foot-sole vibration was removed. Fluctuations with frequencies less than 0.2 Hz were also removed to ensure that a sufficient number of dynamic patterns for the MSE analysis. EMD-filtered time series were then “coarse-grained” to capture system dynamics on different scales of time. This procedure divided the COP time-series into non-overlapping windows of length equaling a scale factor, τ, ranging from 1 to 40 data points, so that the coarse-grained series at the largest scale had 360 data points (i.e., 14400 points/40), which is sufficient to obtain reliable estimates of entropy [6]. We then computed the sample entropy of each coarse-grained time-series by choosing m = 2 and *r* = 15 % based on previous recommendations [6]. Fig. 2 shows the MSE curves generated by plotting sample entropy as a function of time-scale in a representative participant. Finally, the postural sway complexity index was computed as the area under the MSE curve (See Fig. 2, “no vibration” condition), such that larger area reflects higher sample entropy values over multiple time scales and thus, greater complexity.

We previously reported that sub-sensory vibratory noise reduced the average speed and magnitude of postural sway when standing [18]. We therefore included several traditional metrics of postural sway in the current analysis to enable comparison between sway metrics, as well as their relationship to system functionality as measured by the Timed-Up-and-Go (TUG) test (see Statistical Analysis section below). Traditional metrics included postural sway speed (i.e., COP distance traveled divided by duration of one trial) and area (i.e., the area of a confidence ellipse enclosing 95 % of the COP fluctuation).

### Timed Up-and-Go Test (TUG)

Participants completed five TUG trials at each testing session, following the postural control assessment. The TUG test measures the time taken to stand from a chair, walk forward three meters, turn around, walk back and return to a seated position. The average TUG time was computed and used for analysis.

### Statistical analysis

Analyses were performed with JMP Pro 11 software (SAS Institute, Cary NC). First, linear regression analyses were used to examine relationships between baseline foot sole vibration thresholds, postural sway outcomes (i.e., complexity, area, speed) and system functionality (i.e., TUG performance). These models focused on outcomes collected during the control visit only, during which no vibrations were delivered by the insoles. As this visit contained three testing sessions, the within-day testing session (i.e., 1, 2, and 3) was included as a model factor. Second, the effects of sub-sensory vibration on postural sway complexity were assessed using three-way ANOVAs. Model effects included vibration level (0, 70, and 85 %), standing condition (eyes-open, eyes-closed), within-day testing session (1, 2, and 3) and their interactions. Anterioposterior (AP) and mediolateral (ML) complexity were examined in separate models. Tukey’s post-hoc testing was employed to examine mean differences within statistically significant models. Third, linear regression analyses were used to examine the relationships between vibration-induced changes in postural sway outcomes and mobility (TUG times) (e.g., [(Complexity_70 %_ ‐ Complexity_0 %_)/Complexity_0 %_] ∗ 100). Changes induced by vibrations at 70 or 85 % of vibratory threshold were examined separately. The significance level for all analyses was set to *p* < 0.05.

## Results

All 12 participants completed all testing procedures. As previously reported [18], the sensory thresholds of each foot were independently determined multiple times throughout the study. Left and right foot thresholds were moderately correlated (*r*^2^ = 0.14 ~ 0.15, *p* = 0.02 ~ 0.03). The thresholds of each foot remained stable over all obtained assessments (intraclass correlation coefficient >0.99). As such, the average sensory threshold of the right and left foot was computed for each subject and used in all subsequent analyses.

### Relationships between baseline postural control, foot sole vibration threshold and mobility

**Fig. 3:**
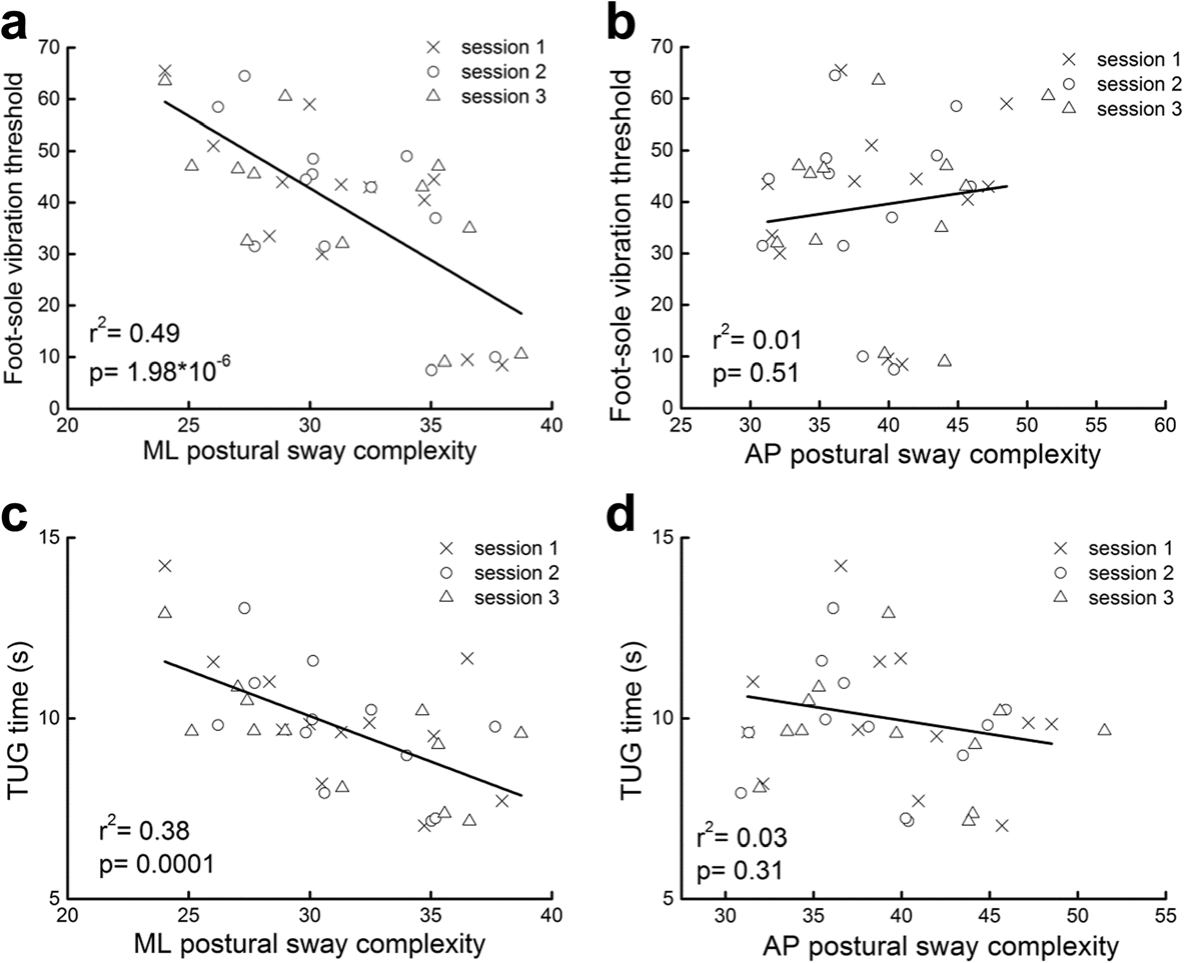
Relationships between postural sway complexity, standing foot sole vibration threshold, and mobility (i.e., timed up-and-go (TUG) time). Scatterplots reflect outcomes acquired during the “baseline” study visit in which no foot sole vibrations were present. Postural sway complexity values were computed from anterioposterior (AP) and mediolateral (ML) center-of-pressure time-series obtained during eyes-open standing. Data from each of the three within-day testing sessions have been included (see Legends). Participants with greater ML postural sway complexity exhibited lower (i.e., more sensitive) foot sole vibratory thresholds, as well as better mobility (i.e., lower TUG times) (**a** and **c**). Within-day testing session did not significantly influence the strength of these correlations. In contrast, neither AP sway complexity (**b** and **d**), nor the traditional measures of sway speed or area (not pictured), were correlated with foot sole somatosensation or mobility

When standing with eyes-open or eyes-closed with vibration at 0 % of threshold (i.e., baseline), neither ML nor AP postural sway complexity correlated with the traditional metrics of postural sway speed or area (*r*2 = 0.001 ~ 0.06, *p* = 0.13 ~ 0.88). Participants with greater ML postural sway complexity exhibited lower bilateral foot sole vibratory sensory threshold (i.e., better somatosensory function, *r*2 = 0.49, *p* < 0.001) and faster TUG times (i.e., greater mobility, *r*2 = 0.38, *p* < 0.001) (Fig. 3a and c). Within-day testing session did not have a significant effect on either aforementioned correlation. There were no significant associations between AP postural sway complexity and somatosensory function (*r*2 = 0.01, *p* = 0.51) or mobility (*r*2 = 0.03, *p* = 0.31) (Fig. 3b and d). Moreover, neither postural sway speed nor area correlated with somatosensory thresholds or mobility (*r*2 = 0.004 ~ 0.1, *p* = 0.1 ~ 0.69).

### Effects of sub-sensory foot sole vibrations on postural control

**Fig. 4:**
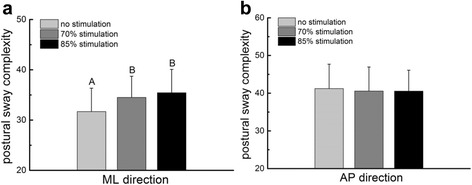
The effects of sub-sensory foot sole vibrations on the complexity of standing postural sway. The influence of vibration level, within-day testing session and standing visual condition on mediolateral (ML) and anterioposterior (AP) postural sway complexity were tested with 3- way repeated-measures ANOVAs. A significant main effect of vibration level was observed for the ML direction (**a**). Tukey’s post-hoc testing revealed that complexity was greater when standing with vibrations applied at both 70 and 85 % of vibratory thresholds (B symbols), as compared to the control condition (A symbol). No other model effects or interactions were present. Postural sway complexity in the AP direction was not influenced by vibration level, testing session or visual condition (**b**). Presented means represent outcomes averaged across within-day testing session and standing visual condition

The effects of sub-sensory foot sole vibrations on the MSE curves generated from the ML postural sway time-series of a representative participant when standing with eyes open are illustrated in Fig. 2. For this participant, sub-sensory vibrations at both 70 and 85 % of sensory threshold resulted in increased entropy across numerous scales of time. Across all participants, the sub-sensory vibratory stimuli increased postural sway complexity (i.e., the area under the MSE curve) *in the ML direction only*. Specifically, a main effect of stimulation level was observed (F_2, 17_ = 12.83, *p* < 0.0001) (Fig. 4a). The main effects of standing visual condition and within-day testing session, as well as all interaction terms, were not significant. For the main effect of stimulation level, post-hoc analyses revealed that 1) postural sway complexity was greater during both 70 and 85 % vibration levels as compared to the control condition, and 2) the effects of both 70 and 85 % sub-sensory vibration levels on complexity were similar to one another. No effects of sub-sensory foot sole vibration were observed on postural sway complexity in the AP direction (Fig. 4b).

### Relationships between vibration-induced changes in postural sway complexity and mobility

**Fig. 5:**
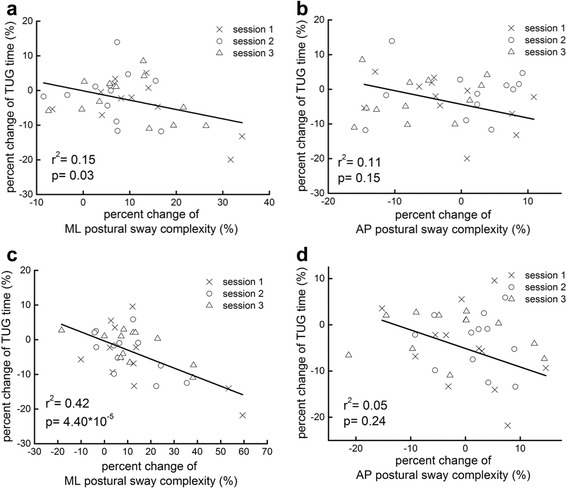
Relationships between sub-sensory vibration-induced changes in postural sway complexity and mobility (i.e., timed up-and-go (TUG) test time) under 70 and 85 % vibration levels. Data from each of the three within-day testing sessions have been included (see Legend). Participants with greater percent increase of ML postural sway complexity improved mobility more (greater percent reduction of time to complete TUG) under both 70 and 85 % vibration levels (**a** and **c**). Within-day testing session did not significantly influence the strength of these correlations. No such associations were observed between changes in anterioposterior (AP) postural sway complexity and TUG performance (**b** and **d**)

In our previous report [18], we demonstrated that foot sole vibrations at both 70 and 85 % of sensory threshold improved TUG performance (i.e. reduced the time taken to complete the test). As sub-sensory foot sole vibrations also increased ML postural sway complexity, regression analyses were completed to determine the relationship between changes in postural sway and TUG performance (as an indicator of mobility and overall functionality of the postural control system). As hypothesized, for both 70 and 85 % stimulation levels, those participants who demonstrated greater percent increases of ML postural sway complexity had greater percent improvement (i.e., reduction) in the time taken to complete the TUG test (*r*2 = 0.15 ~ 0.42, *p* < 0.001 ~ 0.03) (Fig. 5a and c). This relationship was not influenced by within-day testing session. On the other hand, the correlations between the percent change in AP postural sway complexity and the improvement of TUG performance did not reach statistical significance (*r*^2^ = 0.05 ~ 0.11, *p* > 0.15 ~ 0.24) (Fig. 5b and d). Vibration-induced changes in postural sway speed and area were also not related to changes in TUG performance (*r*2 = 0.009 ~ 0.016, *p* = 0.13 ~ 0.47).

## Discussion

The results of this study demonstrate that when standing under normal conditions, healthy older adults who have better foot sole somatosensory function when standing tend to exhibit greater ML postural sway complexity. Moreover, those with greater ML postural sway complexity perform better on the TUG. Vibratory noise applied to the foot soles at amplitudes of both 70 and 85 % of the sensory threshold significantly augmented the physiologic complexity of ML (but not AP) postural sway dynamics in older adults. Importantly, this vibration-induced increase in physiologic complexity correlated with improvement in TUG performance. Together, these results provide novel evidence that the physiologic complexity of postural sway dynamics in older adults may be augmented by sub-sensory foot sole vibrations and moreover, may serve as a marker of overall postural control system functionality.

Lipsitz & Goldberger [20] originally proposed the “complexity theory of aging,” which states that biological aging and disease reduce system functionality by diminishing the quantity and/or quality of the inputs and regulatory elements that control system behavior over time. Moreover, these physiologic changes can be indirectly observed as a loss of physiological complexity—or information content—in the dynamics of that system’s behavior under basal or “free-running” conditions. When standing, the feet are the only points of contact with the external environment. Standing postural control is thus critically dependent upon the capacity of the peripheral nervous system to detect characteristics of the foot-ground interaction and relay this information to the central nervous system. Manor et al. [10] demonstrated that aging-related foot sole somatosensory impairment, as measured by a non-weight-bearing monofilament test on the foot soles, was linked to diminished standing postural sway complexity as well as the ability to adapt one’s posture to cognitive stressors. Here, we observed that those with better standing vibratory sensation exhibit greater postural sway complexity, thus providing first-of-its-kind evidence that standing postural sway complexity is dependent upon *weight-bearing* foot sole somatosensory function in healthy older adults.

Costa et al [16] previously reported that random vibrations applied to the foot sole at 90 % of sensory threshold increased the MSE-derived complexity of standing postural sway in healthy older adults and those with a history of falls. In the currently study, we have demonstrated that this vibratory noise increases ML standing sway complexity when delivered at amplitudes as low as 70 % of sensory threshold. Moreover, the observed augmentation of standing sway complexity and mobility was still present after participants wore the insoles and received vibratory stimulation throughout the entire study visit. Future research is warranted to determine the longer-term therapeutic efficacy of sub-sensory vibratory noise delivered via a shoe insole in larger populations of older adults.

In the current study, the vibration-induced increase of postural sway complexity (at both 70 and 85 % of sensory threshold) correlated with the amount of vibration-induced improvement in TUG performance. In other words, the degree of increase in the physiologic complexity of postural sway was directly correlated with improved functionality. In contrast, no such relationship was observed between changes in the traditional parameters of postural sway speed and area and changes in TUG performance. Together, these results suggest that traditional metrics, which reflect standing postural control system dynamics on only a single temporospatial scale, are not necessarily reflective of one’s ability to complete dynamic tasks such as the TUG [5, 21]. The complexity of standing postural sway fluctuations, on the other hand, appears to sensitively capture the unique multi-scale regulation that enables such functionality.

It is of note that sub-sensory vibratory noise was applied to the feet while participants were standing on the force plate. The addition of this noise may have therefore directly influenced acquisition of the postural sway (i.e., center-of-pressure) time series. Costa et al. [22] showed previously that the complexity of uncorrelated white noise as measured by MSE is significantly lower than other kinds of noise, such as correlated (1/f) noise. Moreover, that study demonstrated the direct superposition of a white noise signal on a non-linear biological or physiological signal either had no effect or *decreased* the complexity of those signals. The observed *increase* in postural sway complexity with the addition of sub-sensory noise is thus notable and likely not a measurement artifact. Instead, it more likely stems from the stochastic resonance phenomenon and a related enhancement of sensory input from the foot soles to the postural control system. Specifically, it is believed that this type of the vibratory noise partially depolarizes mechanoreceptor membranes within the foot soles, thus increasing their likelihood of firing [23] [24]. Consequently, vibration-induced facilitation of neuronal excitability enables the affected neurons to fire in response to relatively smaller external stimuli, thereby enhancing the amount of meaningful input to the system.

In the present study, vibration-induced increases in postural sway complexity were only observed in the ML direction, and not the AP direction. This result suggests that the dynamics of ML sway are particularly sensitive to changes of foot sole somatosensation. This observation is supported by previous findings that chronic impairment of foot-sole sensation, such as that associated with diabetic peripheral neuropathy, is particularly disruptive to postural control in the ML direction during both standing [25] and walking [26]. Moreover, Bernard-Demanze et al. [27] demonstrated that the acute alteration of somatosensation, as induced by a tactile plantar stimulation of 5 Hz over sensory threshold, reduced the magnitude of sway, but only in ML direction, in older adults with foot sole sensory impairment. Moreover, the observed improvements in the control of posture within the ML plane may be particularly meaningful, as a loss of lateral stability is particularly associated with falling [28].

## Conclusions

The current study was based upon a cohort of older adults that was predominantly female and free of major disease and disability. Future work is therefore warranted to examine the effects of sub-sensory vibratory noise stimulation on postural sway complexity and system functionality in larger samples of men and women, as well as patients suffering from neurological diseases such as peripheral neuropathy. While the TUG test is a widely accepted test of mobility and physical function [29], additional work is needed to examine the relationship between postural sway complexity and postural control system functionality as measured by the ability to adapt and respond to physical, cognitive and other stressors. Nevertheless, the present study suggests that vibratory noise applied to the foot soles when standing may improve the functionality of the postural control system in older adults.

## Abbreviations

AP: anteroposterior
ML: mediolateral
MSE: multiscale entropy
TUG: timed up and go
